# Genetic regulation of the *ompX* porin of *Salmonella* Typhimurium in response to hydrogen peroxide stress

**DOI:** 10.1186/s40659-022-00377-3

**Published:** 2022-02-22

**Authors:** A. C. Briones, D. Lorca, A. Cofre, C. E. Cabezas, G. I. Krüger, C. Pardo-Esté, M. S. Baquedano, C. R. Salinas, M. Espinoza, J. Castro-Severyn, F. Remonsellez, A. A. Hidalgo, E. H. Morales, C. P. Saavedra

**Affiliations:** 1 Laboratorio de Microbiología Molecular, Departamento de Ciencias de La Vida, Facultad de Ciencias de la Vida, Universidad Andres Bello, Santiago, Chile; 2 Laboratorio de Microbiología Aplicada Y Extremófilos, Facultad de Ingeniería Y Ciencias Geológicas, Universidad Católica 83 del Norte, Antofagasta, Chile; 3 Centro de Investigación Tecnológica del Agua en El Desierto (CEITSAZA), Universidad Católica del Norte, Antofagasta, Chile; 4 Laboratory of Molecular Pathogenesis and Antimicrobials, Escuela de Química Y Farmacia, Facultad de Medicina, Universidad Andres Bello, Santiago, Chile

**Keywords:** *ompX*, Translational regulation, Transcriptional regulation, H_2_O_2_ stress

## Abstract

**Background:**

*Salmonella* Typhimurium is a Gram-negative pathogen that causes a systemic disease in mice resembling typhoid fever. During its infective cycle, *S.* Typhimurium is phagocytized by macrophages and proliferates inside a *Salmonella*-containing vacuole where *Salmonella* is exposed and survives oxidative stress induced by H_2_O_2_ through modulation of gene expression. After exposure of *Salmonella* to H_2_O_2_, the expression of the porin-encoding gene *ompX* increases, as previously shown by microarray analysis. Expression of *ompX* mRNA is regulated at a post-transcriptional level by MicA and CyaR sRNAs in aerobiosis. In addition, sequence analysis predicts a site for OxyS sRNA in *ompX* mRNA.

**Results:**

In this work we sought to evaluate the transcriptional and post-transcriptional regulation of *ompX* under H_2_O_2_ stress. We demonstrate that *ompX* expression is induced at the transcriptional level in *S*. Typhimurium under such conditions. Unexpectedly, an increase in *ompX* gene transcript and promoter activity after challenges with H_2_O_2_ does not translate into increased protein levels in the wild-type strain, suggesting that *ompX* mRNA is also regulated at a post-transcriptional level, at least under oxidative stress. In silico gene sequence analysis predicted that sRNAs CyaR, MicA, and OxyS could regulate *ompX* mRNA levels. Using rifampicin to inhibit mRNA expression, we show that the sRNAs (MicA, CyaR and OxyS) and the sRNA:mRNA chaperone Hfq positively modulate *ompX* mRNA levels under H_2_O_2_-induced stress in *Salmonella* during the exponential growth phase in Lennox broth.

**Conclusions:**

Our results demonstrate that *ompX* mRNA is regulated in response to H_2_O_2_ by the sRNAs CyaR, MicA and OxyS is *Salmonella* Typhimurium.

**Supplementary Information:**

The online version contains supplementary material available at 10.1186/s40659-022-00377-3.

## Introduction

Every year, *Salmonella enterica* causes around 1800 food-borne illness cases in the United States, resulting in about 200 hospitalizations. Most people develop diarrhea, fever, and abdominal pain 12 to 72 h after infection (CDC, *Salmonella*). During infection, *Salmonella* survives within innate immune host cells, including macrophages [37], where bacteria are exposed to adverse conditions that limit its survival. Reactive Oxygen Species (ROS) are the most harmful of these conditions [20]. ROS generated by phagocytic cells, particularly superoxide anions (O_2_^−^) and hydrogen peroxide (H_2_O_2_), target bacterial components such as proteins, membranes, and nucleic acids [21].

During its infective cycle, internalized *Salmonella* faces H_2_O_2_ and other toxic molecules that enter the bacterium through the outer membrane ([40], Faucher et al. 2006). Studies in *Escherichia coli* and *Saccharomyces cerevisiae* show that H_2_O_2_ cannot freely diffuse across membranes [41, 43]. Evidence from *Salmonella* Typhimurium (*S.* Typhimurium) indicates that ROS are channeled through porins such as OmpW and OmpD, which are down-regulated under oxidative stress *in-vitro*, highlighting their importance for survival [7, 31]. To survive the microenvironment inside the phagosome, *Salmonella* must finely-tune gene expression [16, 35, 36], including the expression of those genes that encode outer membrane proteins, which results in minimizing the influx of ROS [7, 10, 31]. However, a microarray analysis of the transcriptomic profile of *S.* Typhimurium has shown that the expression of the gene that codifies the porin *ompX* was 3.65 times greater under stress conditions compared to control settings [32], suggesting a role for higher level regulation during H_2_O_2_-induced stress for the modulation of the permeability of the outer membrane (Calderon et al. 2011, [32]).

OmpX is a small porin composed of eight antiparallel strands in a barrel conformation [47], that is involved in the responses to H_2_O_2_ stress, host invasion, iron homeostasis, and recognition of bacteria by the host’s adaptive immune response [9, 24, 25, 28, 29]. *ompX* gene expression is regulated at various levels; transcription is increased by overexpression of MarA in *Enterobacter aerogenes* [3, 14] and by H_2_O_2_ exposure in uropathogenic *Escherichia coli* [6].

Mecsas et al. [30] identified two promoters and a rho-terminator in the *ompX* gene in *Escherichia coli*. One of the promoters is bona fide, with sigma 70 boxes (50 bp upstream of the ATG of *ompX* gene). However the authors proposed that the other promoter (221 bp upstream of the ATG of *ompX*) is not activated by sigma 70 under the experimental conditions tested, and could require a supercoiled template, another form of the RNA polymerase or additional transcription factors to promote transcription. The authors suggest that expression of the *ompX* gene is induced under basic pH conditions via the second promoter (221 bp upstream of the ATG), but did not show in vivo evidence to demonstrate promoter activity.

At the post-transcriptional level, *ompX* is regulated by a group of non-coding small RNAs (sRNAs) which modulate the production of the protein and are implicated in a broad array of pathways, including carbon metabolism, iron homeostasis, quorum sensing, biofilm biosynthesis, and stress responses, among other functions [39]. Previous studies suggest that in *Escherichia coli* grown in rich media (Lennox broth), *ompX* mRNA is targeted by the sRNAs MicA and CyaR [22]. During oxidative stress conditions produced by H_2_O_2_, only OxyS sRNA was implicated in the post-transcriptional response, and the main mRNAs regulated by this OxyS encode for proteins required for oxidative stress resistance in *Escherichia coli* [1]. Additionally, the sRNAs CyaR, MicA and OxyS are regulated by the action of Hfq, a sRNA:mRNA chaperone, which stabilizes this interaction, promoting the degradation by RNAses or the translation of the transcript [22, 51], which is also required for regulation under diverse stress conditions [42].

In this work, we investigated *ompX* expression under hydrogen peroxide stress at the transcriptional and post-transcriptional level in *S.* Typhimurium 14028 s. Our results show that *ompX* transcript levels increase under peroxide stress, yet there are no changes at the protein level under the same conditions. Therefore, we hypothesize that MicA, CyaR and OxyS could play relevant roles in the regulation of the translation of *ompX* mRNA under peroxide stress.

## Materials and methods

### Bacterial strains and growth conditions

The bacterial strains and plasmids included in this study are listed in Additional file 1: Table S1. Bacteria were routinely grown in Lennox Broth (LB) at 37ºC supplemented, when necessary, with ampicillin (0.1 mg/ml), chloramphenicol (0.02 mg/ml), or kanamycin (0.05 mg/ml). Cells grown to OD_600_ ≈ 0.4 were treated with 2 mM H_2_O_2_ in LB.

### Construction of chromosomal gene fusions with pSUB11 plasmid

The *ompX::3xflag* strain was constructed as described by Uzzau et al. [45] by fusing the *3xflag* epitope with the *ompX* gene. The primers were designed with 40 homology bases corresponding to the coding regions of the gene and a region immediately downstream to amplify the pSUB11 plasmid (Additional file 2: Table S2). The PCR products were used to transform electrocompetent 14028 s cells carrying plasmid pKD46. Fusion was confirmed by PCR. The 3xFlag-fusion protein was detected by immunoblotting using an anti-FLAG M2 monoclonal antibody (Sigma) and peroxidase-conjugated anti-mouse IgG (Sigma). Proteins were purified and detected as described elsewhere [18].

### Construction of a GFP-transcriptional fusion of *ompX*

*ompX* promoter activity was evaluated by cloning the *ompX* promoter into the pGLO vector (Biorad). For this purpose, 375 bp of the *ompX* promoter were amplified by PCR (primers Prom_*ompX_*-1R and Prom_*ompX*_-375F, Additional file 2: Table S2), and the amplicon and pGLO plasmid were digested using the restriction enzymes *Bmt*I and *Age*I for 1 h at 37 °C. Products were purified using the High Pure PCR Template Kit (Roche) following the manufacturer's instructions. The PCR products were ligated to the digested pGLO plasmid using T4 ligase at 4 °C overnight. Electrocompetent *E. coli* TOP10 cells were then transformed with the resulting plasmid, denominated pGLO_*ompX*. The presence and orientation of the *ompX* promoter was verified by PCR, then the pGLO_*ompX* plasmid was purified using the High Pure Plasmid Isolation Kit (Roche) and transformed into electrocompetent *S.* Typhimurium 14028 s cells. Finally, strains carrying the pGLO_*ompX* and the pGLO plasmids were used to measure GFP fluorescence under peroxide stress after 20 min of treatment.

### Reporter activity

Strains carrying the plasmids pGLO and pGLO_*ompX,* were grown to OD_600_ ≈ 0.4, centrifuged at 4400 rpm for 10 min to concentrate the cells, and suspended in 1 ml 20 mM 1X Phosphate Buffered Saline (PBS). One tube was treated with H_2_O_2_ to a final concentration of 2 mM, and a second tube received no treatment (control). Finally, after 20 min of treatment, 300 μl of each sample were used to measure fluorescence (GFP activity) employing a TECAN Infinite 200 PRO Nanoquant (excitation, 395 nm; emission, 509 nm) microplate reader. Emission values were normalized using the optical densities of treated and untreated strains; the measurement time for each was 3 min, for a total time of 45 min. Fluorescence and OD values were measured in triplicate. The specific fluorescence intensity was calculated using the methods of Eiamphungporn et al. [15], where the corresponding OD of the culture was used to normalize the initial and final fluorescence values of the construct and the empty vector. Specific fluorescence was calculated using the equation: (Δfluorescence/ΔOD)_construct_—(Δfluorescence/ΔOD)_empty vector_ for all strains subjected to treatments. All measurements were normalized using the values obtained from the wild-type strain grown under control conditions (no treatment).

### RNA isolation and real-time quantitative PCR

Overnight bacterial cultures grown in LB were diluted (1:100). Cells were then grown to OD_600_≈0.4 and subjected to H_2_O_2_ treatment directly in the medium; one tube remained as an untreated control. After 20 min of incubation with H_2_O_2_, RNA was extracted using the acid–phenol method and the purified RNA was suspended in 30 μl of nuclease-free water. Finally, RNA integrity, quality, and quantity were verified using 1% agarose electrophoresis and A_260_/_280_ ratio. Total RNA was treated with DNase I, and cDNA was synthesized using M-MLV RT (Promega) and random primers following the manufacturer's instructions. cDNA was quantified by qRT-PCR using the primers shown in Additional file 2: Table S2. Relative quantification was performed using the Brilliant II SYBR Green qPCR Master Reagent Kit and the Mx3000P detection system (Stratagene); *talB* gene was used for normalization [5, 35]. Amplification efficiency was calculated using a standard curve constructed by amplifying serial dilutions of RT-PCR products for each gene. These values were used to obtain the fold-change in the expression of the gene of interest.

### Rifampicin assay

To evaluate the influence of the sRNA on *ompX* mRNA, we performed a rifampicin assay over time. Briefly, the strains (wild-type, Δ*micA*, Δ*cyaR*, Δ*oxyS* and Δ*hfq*) were grown in LB broth at 37ºC with constant agitation. Once the cultures reached OD_600_ ≈ 0.4 they were divided into two equal batches, one being the control whilst the other was treated with 2 mM H_2_O_2_. Then, at the predetermined time points (0, 5, 10, 15 and 20 min), 10 ml of culture were subjected to RNA extraction, cDNA preparation and the determination of *ompX* and *talB* expression by qRT-PCR, as described above. We performed two approaches: a rifampicin absent (–RIF) and rifampicin present (+ RIF) assay. For the rifampicin present assay, we added rifampicin (20 µg/ml) when the cells originally reached an OD_600_ ≈ 0.4.

### Colony-forming units

To determine the colony-forming units (CFUs), *S.* Typhimurium 14028 s strains (wild-type, Δ*micA*, Δ*cyaR,* Δ*oxyS,* Δ*ryhB* and Δ*hfq*) were grown to OD_600_ ≈ 0.4 in LB using the corresponding antibiotic selection. Except in controls, H_2_O_2_ was added to a final concentration of 2 mM, and incubation took place with agitation at 37 °C for 20 min. After incubation, all strains were serially-diluted in PBS and spotted on LB agar. CFU were counted the following day, and the assay was replicated 5 times. We calculated percentage of survival using arbitrarily the wild-type strain grown under control conditions as 100% of survival.

### Total intracellular ROS determination

To determine total intracellular ROS, the H_2_DCFDA (Sigma-Aldrich) probe was used. Briefly, *S.* Typhimurium 14028 s strains (wild-type, Δ*micA*, Δ*cyaR,* Δ*oxyS,* Δ*ryhB* and Δ*hfq*) were grown to OD_600_ ≈ 0.4. Except in controls, H_2_O_2_ was added to a final concentration of 2 mM, and incubation took place with agitation at 37 °C for 20 min. After incubation, 300 µl of bacterial cultures were withdrawn in triplicate and placed in a 96-well microplate. Fluorescence was measured every 5 min, for a total of 45 min (excitation, 480 nm; emission, 520 nm). Total intracellular ROS were calculated as: (Δfluorescence/time)/ΔOD_600_. The final concentration of the probe was 10 µM in DMSO.

### In silico gene sequence evaluation

To evaluate whether OxyS has binding sites on the *ompX* mRNA, we performed an *in-silico* analysis using the tool IntaRNA [4, 49] and the nucleotide sequences of the *ompX* mRNA and OxyS sRNA.

### Statistical analysis

Gene-by-gene comparisons were performed as individual experiments for each time point using one-way ANOVAs with α = 0.05. Statistical analyses were performed with the Bonferroni correction comparing individual mutant strains with the wild-type strain. We used the Prism 7 software to perform all the data analysis.

## Results

### H_2_O_2_ stress increases levels of *ompX* mRNA but not of OmpX protein.

**Fig. 1:**
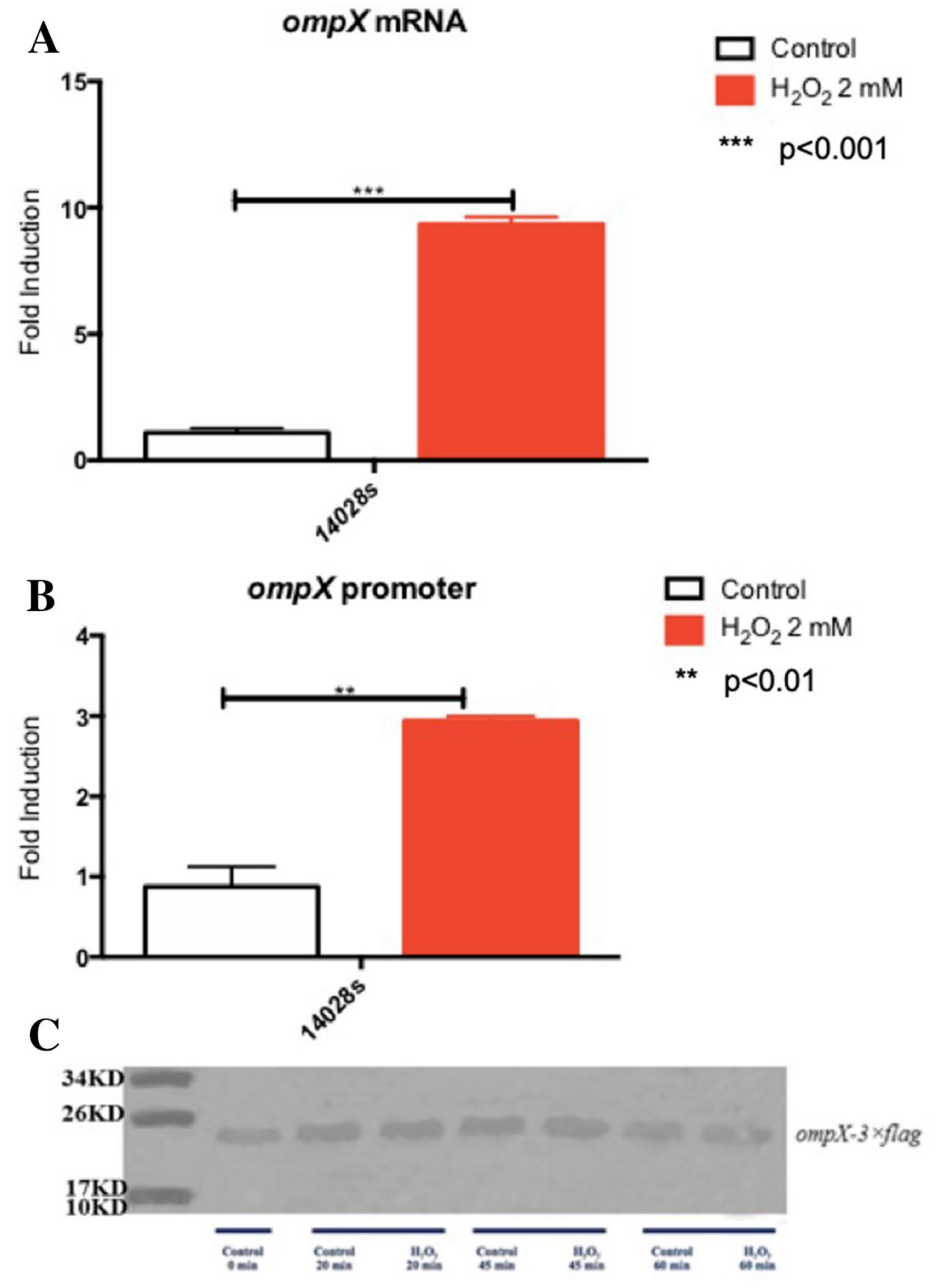
Analysis of *ompX* expression. **A** mRNA level of *ompX* in the wild-type strain of *S.* Typhimurium 14028 s. **B** Fluorescence activity of GFP under the control of the *ompX* promoter (-353 to -1) in the wild-type *S*. Typhimurium background. **C** Immunodetection of the OmpX::3xFlag protein, measured after exposure of the strain to 2 mM H_2_O_2_ for 20, 45 and 60 min. The control received no treatment. Ten µg of total proteins were loaded. White bars represent the control (no treatment), and red bars represent cells treated with 2 mM H_2_O_2_. The graph represents the average of 3 independent experiments (mean ± SD or SE?)

Previously, our group reported that *ompX* transcript levels increased under peroxide stress [32]. To further evaluate the mechanism of *ompX* regulation under these conditions, we determined *ompX* transcript levels as well as promoter activity under H_2_O_2_-induced stress. Promoter activity was measured using the two putative promoters identified by Mecsas et al. [30]. As shown previously, *ompX* transcript levels increased eight-times after exposure to hydrogen peroxide (Fig. 1A), while promoter activity increased almost three-fold (Fig. 1B).

To investigate whether the observed increase in *ompX* mRNA and promoter activity levels correlated with higher protein levels, we measured the OmpX protein by immunoblot. Unexpectedly, OmpX protein levels were not affected by the peroxide treatment (Fig. 1C). These results suggest that OmpX production may be subjected to post-transcriptional regulation under these stress conditions. Previous evidence suggests that sRNAs such as MicA, RybB, CyaR and Hfq regulate this porin post-transcriptionally under standard growth conditions. For instance, CyaR contains a C‐rich apical loop that sequesters the Shine–Dalgarno sequence of *ompX* mRNA and inhibits translation initiation [11, 17, 23, 34]. However, to our knowledge, the specific mechanism that occurs under ROS-enriched conditions has not been elucidated and should take into consideration the role of molecules that are targeted by hydrogen peroxide and that can potentially lose function as a consequence of oxidative damage.

### *ompX* is regulated at the post-transcriptional level in response to H_2_O_2_

**Fig. 2:**
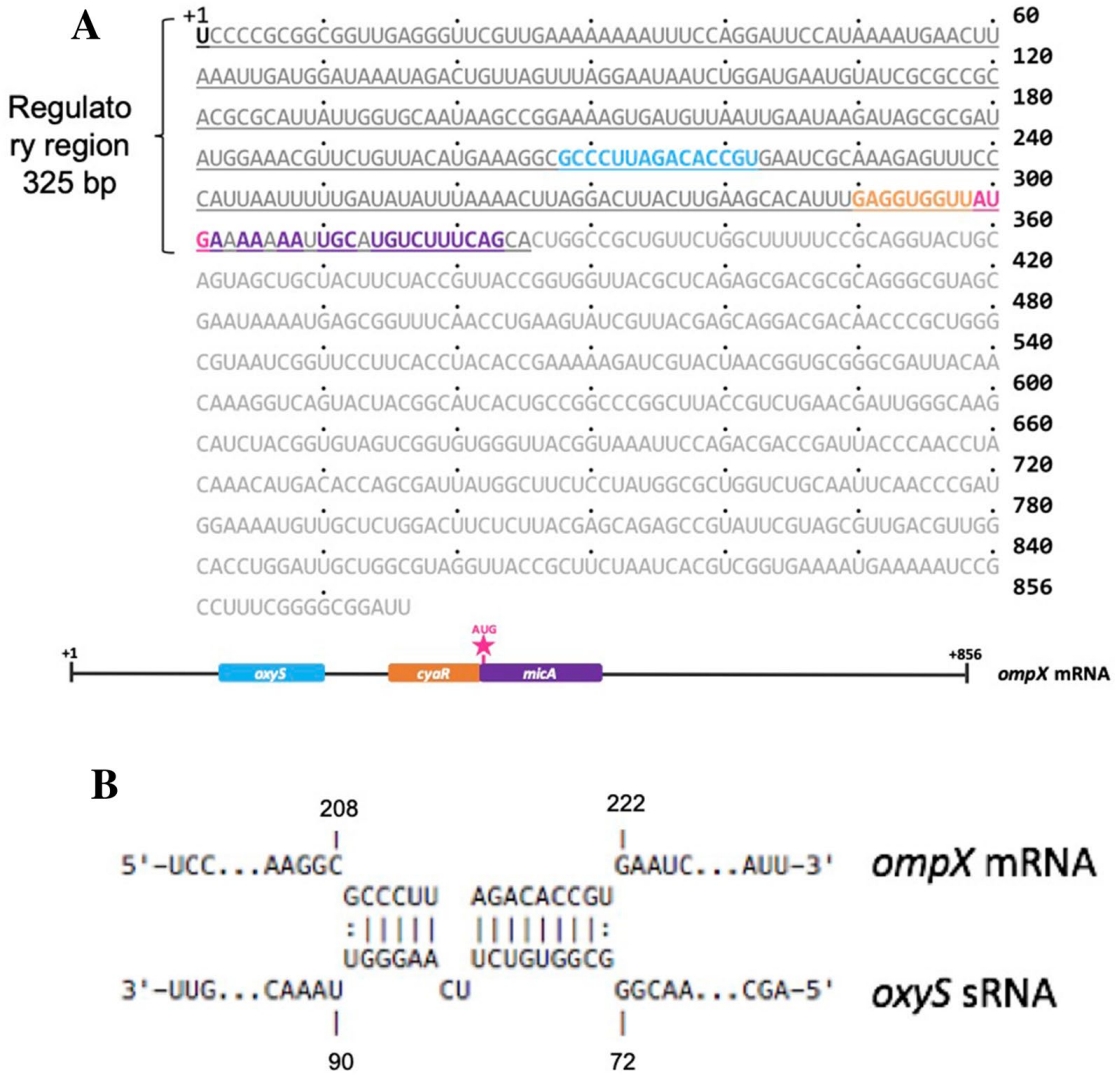
Regulation model of *ompX* mRNA under H_2_O_2_ stress. **A** Binding sites of the *oxyS, micA* and *cyaR* sRNAs in the 325 bp-regulatory region of *ompX* mRNA (the whole transcript is 836 bp long) under oxidative stress (*oxyS* in light blue, *cyaR* in orange, and *micA* in purple, translation start site in pink)*.* **B** Schematic representation of the proposed interaction of *oxyS* sRNA with the *ompX* mRNA*.* The prediction was made using IntaRNA [4, 49],numbers indicate positions in the *ompX* mRNA and the *oxyS* sRNA sequence, respectively

In *Escherichia coli*, *ompX* mRNA is regulated at the post-transcriptional level by CyaR and MicA [22]. In silico analysis revealed the presence of a putative interaction domain between OxyS and *ompX* mRNA (Fig. 2), suggesting that OxyS could participate in some way in the regulation of *ompX* mRNA under oxidative stress.

**Fig. 3:**
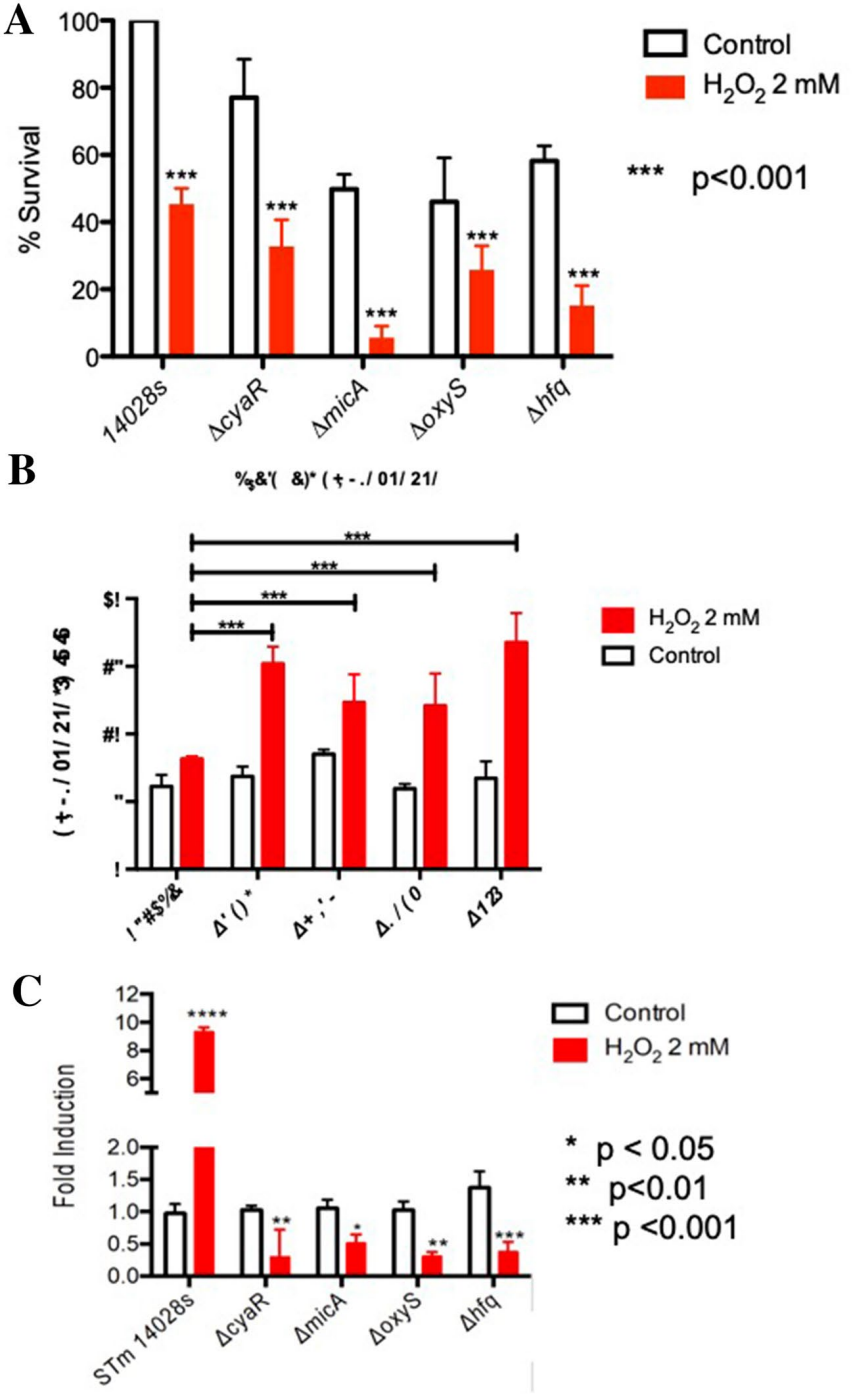
Role of *cyaR*, *micA* and *oxyS* sRNAs in oxidative stress.** A** Percentage of survival based on Colony Forming Units (CFU) of the sRNA-mutants under peroxide stress compared to *S.* Typhimurium 14028 s under control conditions. **B** ROS accumulation of the single sRNA mutants under peroxide stress. **C** *ompX* mRNA levels in wild-type, Δ*cyaR,* Δ*micA,* Δ*oxyS*, Δ*hfq* and Δ*ryhB* strains of *S.* Typhimurium 14028 s were measured by qRT-PCR. Strains were exposed to 2 mM H_2_O_2_ for 20 min (red bars); the control had no treatment (white bars). The graph represents the average of 5 independent experiments (mean ± SD or SE?)

Given their previously described roles in model bacteria such as *Escherichia coli*, we determined whether CyaR, MicA, or OxyS play relevant roles during peroxide stress in *S*. Typhimurium by examining bacterial survival and intracellular ROS accumulation. Bacterial strains with the deletion of each sRNA show decreased survival in comparison to the wild-type control, especially after deletion of *micA* (Fig. 3A). Furthermore, intracellular ROS accumulation is also greater in mutant strains (Fig. 3B), suggesting that this stressor has an even more detrimental effect on the overall survival of the bacteria when the function of these sRNAs is absent.

Moreover, the amount of *ompX* transcript significantly increases in the wild-type strain after ROS-induced stress, but not in the isogenic mutant strains for each sRNA (Fig. 3C), suggesting a role in the regulation of the porin during ROS-resistance. The results suggest that CyaR, MicA, and OxyS are required to maintain *ompX* levels under oxidative stress 20 min post-treatment, indicating that at least partially, this mechanism of survival to ROS-induced stress is MicA-, CyaR- and OxyS-dependent.

**Fig. 4:**
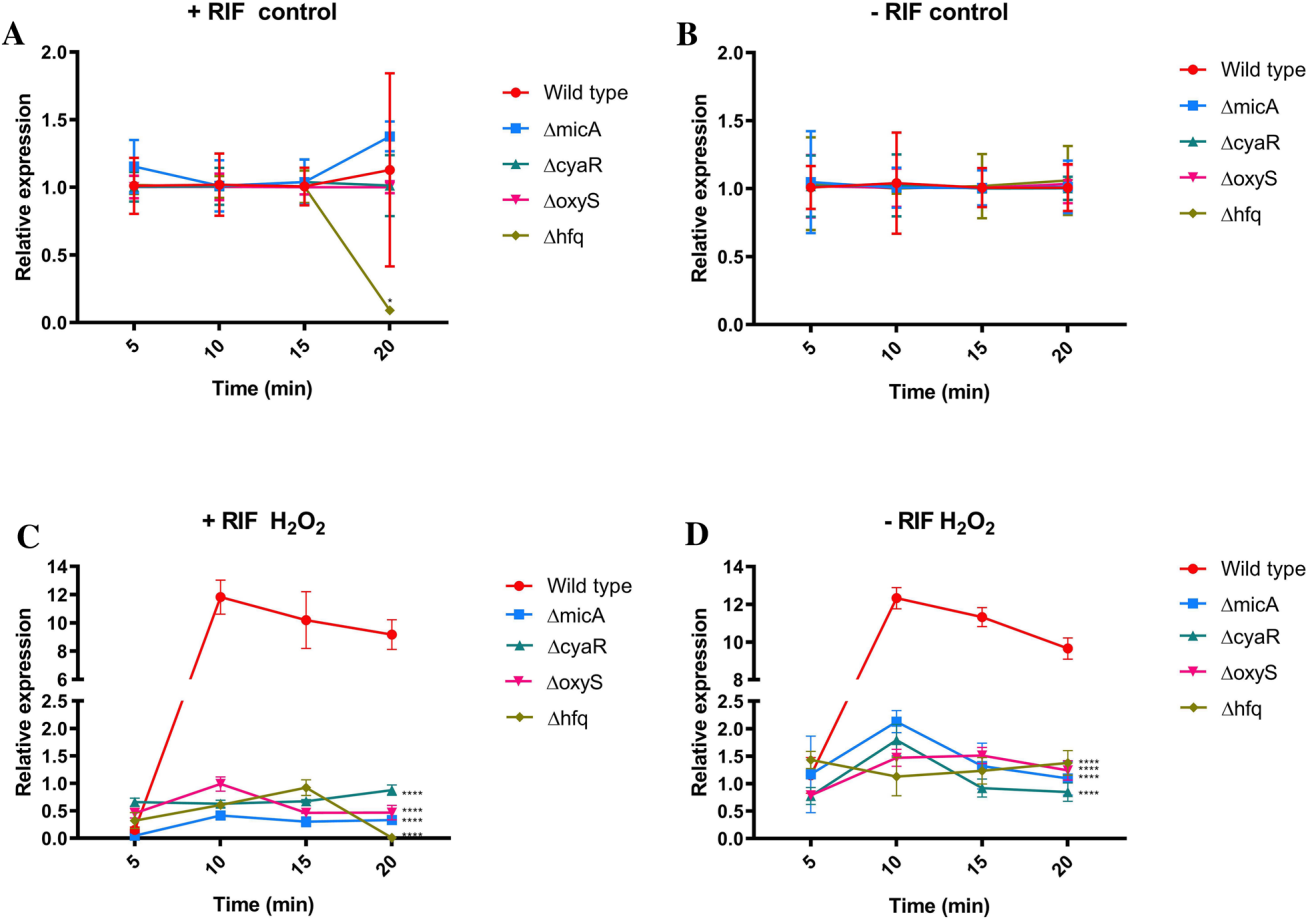
Effect of rifampicin on *ompX* mRNA under H_2_O_2_-induced stress. *ompX* mRNA levels were measured by qRT-PCR in the following strains: wild-type, Δ*micA,* Δ*cyaR,* Δ*oxyS* and Δ*hfq.* When cultures reached an OD_600_ ≈ 0.4, a pulse of rifampicin (20 µg/ml) was added or not (+ RIF red circles or –RIF inverted blue triangles) to the culture at time 0 min. At each time point, RNA was isolated and subjected to qRT-PCR as described in Materials and Methods. Post-test Bonferroni: *p < 0.05, ***p < 0.01 and ****p < 0.0001, (mean ± SD or SE?)

To evaluate the post-transcriptional regulation of the *ompX* mRNA in response to peroxide stress, we assessed *ompX* expression after treating bacteria with rifampicin during 20 min and measured expression at 5 min intervals. Rifampicin is an antibiotic that affects the RNA polymerase, specifically the elongation process; therefore, changes in mRNA content after inhibiting mRNA production are due to post-transcriptional processes [48]. This approach enabled us to generate more evidence regarding the post-transcriptional regulation of OmpX under ROS-related stress, by using an antibiotic that inhibits RNA polymerase and observing changes in mRNA production associated with post-transcriptional processes. Also, as controls we measured the effect of rifampicin on the wild-type strain and found that there is a significant decrease in the stability and amount of *ompX* expression (Additional file 3: Fig. S1). Moreover, we found that adding 5 µg/mL rifampicin reduced the levels of transcripts (Additional file 4: Fig. S2), validating the use of this approach to measure *ompX* expression under ROS stress. The results indicate that there is no difference regarding stability and amount of *ompX* in the control conditions with (Fig. 4A) or without (Fig. 4B) the antibiotic treatment. However, once bacteria were under ROS-induced stress, we found a statistically significant decrease in the amount of *ompX* transcripts in all mutant strains compared to the wild-type strain (Fig. 4C). Using the rifampicin treatment, we determined that the decreased amount of transcript of the mutant strains compared to the wild-type is a consequence of the activity of the sRNA during post-transcriptional regulation, as the levels detected in the wild-type strains are significantly higher than those in the strains that lack the genes for the different sRNA tested.

These findings indicate that MicA, CyaR, OxyS and Hfq exert their regulatory functions once the ROS-induced stress is signaled to the cell inducing the expression of this small porin. Our results allow us to suggest that these three sRNAs (MicA, CyaR and OxyS) are required to stabilize the *ompX* mRNA in an Hfq-dependent manner, when *Salmonella* faces hydrogen peroxide-induced stress.

## Discussion

Previous studies showed that increased OmpX expression alters the levels of other outer membrane porins, such as OmpC and OmpF, and increases sigma E (σ^E^)-containing RNA polymerase activity [30]. Our results show that under H_2_O_2_-induced stress, the levels of *ompX* transcript increased as well as its promoter activity (Fig. 1A, B), yet there was no increase in OmpX protein levels (Fig. 1C), suggesting that *ompX* is regulated post-transcriptionally. We found that *ompX* mRNA is regulated by MicA, OxyS, and CyaR in response to H_2_O_2_. Furthermore, we speculate that lower *ompX* mRNA levels, observed in response to ROS, result from inhibition in translation, given that mRNA stability decreases when translation initiation is halted [8, 13].

Supporting this view, MicA and OxyS affected OmpX protein levels (Additional file 5: Fig. S3). MicA expression, induced by various stresses (some of which can be found inside the phagosome), is regulated by σ^E^, which plays a key role in the oxidative stress response [19, 20, 27, 46] inhibiting the expression of *ompW* and *phoP* [46]. Srikumar et al. [44] demonstrated that MicA and OxyS are upregulated in *Salmonella* during infection of murine macrophages. The former (MicA) can adopt various conformations, and dimerization inhibits its function [46]. OxyS regulates *rpoS*, encoding an alternative sigma factor required for responses to low temperatures, osmotic shock, and membrane stress [38] as well as *fhlA*, encoding a protein involved in metabolic stress adaptation [2]. *cyaR* expression is slightly induced in *Yersenia pestis* exposed to peroxide stress [50] and is regulated under nutrient deprivation, a condition that can be found in *Salmonella*-containing vacuoles (SCV; [11, 17, 20]). All this data supports our hypothesis that these sRNAs participate in ROS response and could function to modulate positively *ompX* transcripts.

Previous studies suggested that protein production depends on three factors: transcription rate, mRNA degradation and mRNA concentration, factors that depend on the cell status [12]. When mRNA levels increase as a function of increased transcription, mRNA stability is strongly affected. Thus, mRNA stability depends on mRNA concentration [33], among other factors. In the same study, the authors suggest that mRNA levels must reach an equilibrium imposed by the condition and energetic status of the cell, a way to control and reduce the energetic cost associated with the production of new proteins [33].

OmpX is essential for invasion of various bacterial species such as *Enterobacter, Yersinia,* and *Cronobacter* [9, 24, 25], and for iron homeostasis in *E. coli* [28]. Therefore, fine regulation of OmpX under oxidative stress could be an adaptation to the stress encountered inside macrophages, where the bacteria attempt to balance the influx of essential metabolites with the uptake of ROS through this porin. Recently, it has been found that the OmpX porin is involved in biofilm formation and curli production [26]. Thus, these critical roles require fine regulation in response to specific conditions that enable bacteria to adapt efficiently. Further studies are necessary to determine the specific role of this protein during the oxidative stress, including other strategies such as antisense RNA, proteomic and in silico analyses of the structural relationships among all molecules.

## Conclusions

Our results show that *ompX* mRNA is regulated in response to H_2_O_2_ by the sRNAs CyaR, MicA and OxyS. These data represent a step forward in this area and provide additional insights into the complex regulation of *ompX* in response to H_2_O_2_-induced stress, a physiologically relevant condition encountered by *S.* Typhimurium during its infection cycle.

## Supplementary Information

**Additional file 1: Table S1.** Bacterial strains and plasmids used in this work.

**Additional file 2: Table S2.** Primers used in this work.

**Additional file 3: Figure S1.** Effect of rifampicin on *ompX* mRNA under H_2_O_2_-induced stress in the wild-type strain. *ompX* mRNA levels were measured by qRT-PCR. When cultures reached an OD_600_ ≈ 0.4, a pulse of rifampicin (20 µg/ml) was added or not (+ RIF red line or –RIF blue line) to the culture at time 0 min. At each time point, RNA was isolated and subjected to qRT-PCR as described in “Materials and Methods”. Post-test Bonferroni: *p < 0.05, **p < 0.01 and *** p < 0.0001, (mean ± SD or SE?).

**Additional file 4: Figure S2.** Effect of rifampicin concentration on *ompX*. The *ompX* mRNA levels were measured by qRT-PCR. When cultures reached an OD_600_ ≈ 0.4, a pulse of rifampicin (0, 2.5, 5 and 10 µg/mL), was added. At each time point, RNA was isolated and subjected to qRT-PCR as described in Materials and Methods.

**Additional file 5:** **Figure S3.** Production of OmpX::Flag protein in *S.* Typhimurium after exposure to H_2_O_2_. Outer-membrane fractions of each strain were isolated after culture under control or 2 mM H_2_O_2_ treatment (20 min in Lennox broth at 37 ºC). Lanes 1 and 2: *ompX::3xflag* control and H_2_O_2_-treated. Lanes 3 and 4: Δ*cyaR ompX::3xflag* control and H_2_O_2_-treated. Lanes 5 and 6: Δ*micA ompX::3xflag* control and H_2_O_2_-treated. Lanes 7 and 8: Δ*oxyS ompX::3xflag* control and H_2_O_2_-treated. Lanes 9 and 10: Δ*hfq ompX::3xflag* control and H_2_O_2_-treated. Total protein extracts (100 µg) were resolved by SDS-PAGE. The assay shown is representative of three biological replicates.

## Data Availability

All data is available upon request.

## Abbreviations

*ompX*: Outer membrane porin X
mRNA: Messenger Ribonucleic Acid
sRNA: Small Ribonucleic Acid
*S.*Typhimurium: *Salmonella enterica* Serovar Typhimurium
O_2_^−^: Superoxide anion
H_2_O_2_: Hydrogen peroxide
ROS: Reactive Oxygen Species
RIF: Rifampicin
LB: Lennox Broth
OD: Optical density
CFU: Colony forming Units
GFP: Green Fluorescence Protein

## References

[1] Altuvia S, Weinstein-Fischer D, Zhang A, Postow L, Storz G (1997). A small, stable RNA induced by oxidative stress: role as a pleiotropic regulator and antimutator. Cell.

[2] Altuvia S, Zhang A, Argaman L, Tiwari A, Storz G (1998). The *Escherichia coli* OxyS regulatory RNA represses *fhlA* translation by blocking ribosome binding. EMBO J.

[3] Barbosa TM, Levy SB (2000). Differential expression of over 60 chromosomal genes in *Escherichia coli* by constitutive expression of MarA. J Bacteriol.

[4] Busch A, Richter AS, Backofen R (2008). IntaRNA: efficient prediction of bacterial sRNA targets incorporating target site accessibility and seed regions. Bioinformatics.

[5] Cabezas CE, Briones AC, Aguirre C, Pardo-Esté C, Castro-Severyn J, Salinas CR, Baquedano MS, Hidalgo AA, Fuentes J, A., Morales, E. H., Meneses, C. A., Castro-Nallar, E., & Saavedra, C. P. (2018). The transcription factor SlyA from *Salmonella* Typhimurium regulates genes in response to hydrogen peroxide and sodium hypochlorite. Res Microbiol.

[6] Cadieux PA, Burton J, Devillard E, Reid G (2009). *Lactobacillus* by-products inhibit the growth and virulence of uropathogenic *Escherichia coli*. J Physiol Pharmacol.

[7] Calderón IL, Morales E, Caro NJ, Chahúan CA, Collao B, Gil F, Villareal JM, Ipinza F, Mora G, Saavedra CP (2011). Response regulator ArcA of *Salmonella enterica* serovar Typhimurium downregulates expression of OmpD, a porin facilitating uptake of hydrogen peroxide. Res Microbiol.

[8] Carpousis AJ, Luisi BF, McDowall KJ (2009). Endonucleolytic initiation of mRNA decay in *Escherichia coli*. Prog Mol Biol Transl Sci.

[9] de Kort G, Bolton A, Martin G, Stephen J, van de Klundert JA (1994). Invasion of rabbit ileal tissue by *Enterobacter cloacae* varies with the concentration of OmpX in the outer membrane. Infect Immunol.

[10] De la Cruz MÁ, Calva E (2010). The complexities of porin genetic regulation. J Mol Microbiol Biotechnol.

[11] De Lay N, Gottesman S (2009). The Crp-activated small noncoding regulatory RNA CyaR (RyeE) links nutritional status to group behavior. J Bacteriol.

[12] Dressaire C, Picard F, Redon E, Loubière P, Queinnec I, Girbal L, Cocaign-Bousquet M (2013). Role of mRNA stability during bacterial adaptation. PLoS ONE.

[13] Dreyfus M (2009). Killer and protective ribosomes. Prog Mol Biol Transl Sci.

[14] Dupont M, Dé E, Chollet R, Chevalier J, Pagès J-M (2004). *Enterobacter aerogenes* OmpX, a cation-selective channel mar - and osmo-regulated. FEBS Lett.

[15] Eiamphungporn W, Prachayasittikul S, Isarankura-Na-Ayudhya C, Prachayasittikul V (2012). Development of bacterial cell-based system for intracellular antioxidant activity screening assay using green fluorescence protein (GFP) reporter. Afr J Biotech.

[16] Eriksson S, Lucchini S, Thompson A, Rhen M, Hinton JC (2003). Unravelling the biology of macrophage infection by gene expression profiling of intracellular *Salmonella enterica*. Mol Microbiol.

[17] De Lay N, Gottesman S (2009). The Crp-activated small noncoding regulatory RNA CyaR (RyeE) links nutritional status to group behavior. J Bacteriol.

[18] Gil F, Ipinza F, Fuentes J, Fumeron R, Villarreal JM, Aspée A, Mora G, Vásquez CC, Saavedra CP (2007). The *ompW* (porin) gene mediates methyl viologen (paraquat) efflux in *Salmonella enterica* serovar Typhimurium. Res Microbiol.

[19] Gogol EB, Rhodius VA, Papenfort K, Vogel J, Gross CA (2011). Small RNAs endow a transcriptional activator with essential repressor functions for single-tier control of a global stress regulon. Proc Natl Acad Sci.

[20] Hébrard M, Viala JPM, Meresse S, Barras F, Aussel L (2009). Redundant hydrogen peroxide scavengers contribute to *Salmonella* virulence and oxidative stress resistance. J Bacteriol.

[21] Imlay JA (2013). The molecular mechanisms and physiological consequences of oxidative stress: lessons from a model bacterium. Nat Rev Microbiol.

[22] Johansen J, Eriksen M, Kallipolitis B, Valentin-Hansen P (2008). Down-regulation of outer membrane proteins by noncoding RNAs: unraveling the cAMP–CRP- and σE-dependent CyaR–*ompX* regulatory case. J Mol Biol.

[23] Kakoschke TK, Kakoschke SC, Zeuzem C, Bouabe H, Adler K, Heesemann J, Rossier O (2016). The RNA chaperone Hfq is essential for virulence and modulates the expression of four adhesins in Yersinia enterocolitica. Sci Rep.

[24] Kim KP, Choi J, Lim JA, Lee J, Hwang S, Ryu S (2010). Outer membrane proteins A (OmpA) and X (OmpX) are essential for basolateral invasion of *Cronobacter sakazakii*. Appl Environ Microbiol.

[25] Kolodziejek AM, Sinclair DJ, Seo KS, Schnider DR, Deobald CF, Rohde HN, Viall AK, Minnich SS, Hovde CJ, Minnich SA, Bohach GA (2007). Phenotypic characterization of OmpX, an Ail homologue of *Yersinia pestis* KIM. Microbiology.

[26] Li B, Huang Q, Cui A, Liu X, Hou B, Zhang L, Liu M, Meng X, Li S (2018). Overexpression of outer membrane protein X (OmpX) compensates for the effect of TolC inactivation on biofilm formation and curli production in extraintestinal pathogenic *Escherichia coli* (ExPEC). Front Cell Infect Microbiol.

[27] Li J, Overall CC, Johnson RC, Jones MB, McDermott JE, Heffron F, Cambronne ED, Adkins JN (2015). ChIP-seq analysis of the σE regulon of *Salmonella enterica* serovar typhimurium reveals new genes implicated in heat shock and oxidative stress response. PLoS ONE.

[28] Lin X, Wu L, Li H, Wang S, Peng X (2008). Downregulation of Tsx and OmpW and upregulation of OmpX are required for iron homeostasis in *Escherichia coli*. J Proteome Res.

[29] Maisnier-Patin K, Malissard M, Jeannin P, Haeuw JF, Corbière JC, Hoeffel G, Gauchat JF, Nguyen T, Saez JM, Delneste Y (2003). The outer membrane protein X from *Escherichia coli* exhibits immune properties. Vaccine.

[30] Mecsas J, Welch R, Erickson JW, Gross CA (1995). Identification and characterization of an outer membrane protein, OmpX, in *Escherichia coli* that is homologous to a family of outer membrane proteins including Ail of *Yersinia enterocolitica*. J Bacteriol.

[31] Morales EH, Calderón IL, Collao B, Gil F, Porwollik S, McClelland M, Saavedra CP (2012). Hypochlorous acid and hydrogen peroxide-induced negative regulation of *Salmonella enterica* serovar Typhimurium *ompW* by the response regulator ArcA. BMC Microbiol.

[32] Morales EH, Collao B, Desai PT, Calderón IL, Gil F, Luraschi R, Porwollik S, McClelland M, Saavedra CP (2013). Probing the ArcA regulon under aerobic/ROS conditions in *Salmonella enterica* serovar Typhimurium. BMC Genomics.

[33] Nouaille S, Mondeil S, Finoux AL, Moulis C, Girbal L, Cocaign-Bousquet M (2017). The stability of an mRNA is influenced by its concentration: a potential physical mechanism to regulate gene expression. Nucleic Acids Res.

[34] Papenfort K, Pfeiffer V, Lucchini S, Sonawane A, Hinton JC, Vogel J (2008). Systematic deletion of Salmonella small RNA genes identifies CyaR, a conserved CRP-dependent riboregulator of OmpX synthesis. Mol Microbiol.

[35] Pardo-Esté C, Hidalgo AA, Aguirre C, Briones AC, Cabezas CE, Castro-Severyn J, Fuentes JA, Opazo CM, Riedel CA, Otero C, Pacheco R, Valvano MA, Saavedra CP (2018). The ArcAB two-component regulatory system promotes resistance to reactive oxygen species and systemic infection by *Salmonella* Typhimurium. PLoS ONE.

[36] Pardo-Esté C, Castro-Severyn J, Krüger GI, Cabezas CE, Briones AC, Aguirre C, Saavedra CP (2019). The transcription factor ArcA modulates Salmonella’s metabolism in response to neutrophil hypochlorous acid-mediated stress. Front Microbiol.

[37] Pham OH, McSorley SJ (2015). Protective host immune responses to *Salmonella* infection. Future Microbiol.

[38] Repoila F, Majdalani N, Gottesman S (2003). Small non-coding RNAs, co-ordinators of adaptation processes in *Escherichia coli*: the RpoS paradigm. Mol Microbiol.

[39] Richards GR, Vanderpool CK (2011). Molecular call and response: the physiology of bacterial small RNAs. Biochimica Biophysica Acta Gene Regulatory Mechanisms.

[40] Rosenberger CM, Gallo RL, Finlay BB (2004). Interplay between antibacterial effectors: a macrophage antimicrobial peptide impairs intracellular *Salmonella* replication. Proc Natl Acad Sci.

[41] Seaver LC, Imlay J (2001). Hydrogen peroxide fluxes and compartmentalization inside growing *Escherichia coli*. J Bacteriol.

[42] Sittka A, Pfeiffer V, Tedin K, Vogel J (2007). The RNA chaperone Hfq is essential for the virulence of *Salmonella* Typhimurium. Mol Microbiol.

[43] Sousa-Lopes A, Antunes F, Cyrne L, Marinho HS (2004). Decreased cellular permeability to H_2_O_2_ protects *Saccharomyces cerevisiae* cells in stationary phase against oxidative stress. FEBS Lett.

[44] Srikumar S, Kröger C, Hébrard M, Colgan A, Owen SV, Sivasankaran SK, Cameron ADS, Hokamp K, Hinton JC (2015). RNA-seq brings new insights to the intra-macrophage transcriptome of *Salmonella* Typhimurium. PLoS Pathog.

[45] Uzzau S, Figueroa-Bossi N, Rubino S, Bossi L (2001). Epitope tagging of chromosomal genes in *Salmonella*. Proc Natl Acad Sci.

[46] Van Puyvelde S, Vanderleyden J, De Keersmaecker SCJ (2015). Experimental approaches to identify small RNAs and their diverse roles in bacteria - what we have learnt in one decade of MicA research. Microbiology Open.

[47] Vogt J, Schulz GE (1999). The structure of the outer membrane protein OmpX from *Escherichia coli* reveals possible mechanisms of virulence. Structure.

[48] Wehrli W, Knüsel F, Schmid K, Staehelin M (1968). Interaction of rifamycin with bacterial RNA polymerase. Proc Natl Acad Sci U S A.

[49] Wright PR, Georg J, Mann M, Sorescu DA, Richter AS, Lott S, Kleinkauf R, Hess WR, Backofen R (2014). CopraRNA and IntaRNA: predicting small RNA targets, networks and interaction domains. Nucleic Acids Res.

[50] Yan Y, Su S, Meng X, Ji X, Qu Y, Liu Z, Wang X, Cui Y, Deng Z, Zhou D, Jiang W, Yang R, Han Y (2013). Determination of sRNA expressions by RNA-seq in *Yersinia pestis* grown *in vitro* and during infection. PLoS ONE.

[51] Zhang A, Wassarman KM, Rosenow C, Tjaden BC, Storz G, Gottesman S (2003). Global analysis of small RNA and mRNA targets of Hfq. Mol Microbiol.

