# Supplementary material for: Genetic regulation of the ompX porin of Salmonella Typhimurium in response to hydrogen peroxide stress
Source: Biol Res. 2022 Feb 22;55:8. doi: 10.1186/s40659-022-00377-3 (PMC8862304; doi:10.1186/s40659-022-00377-3)
Supplement: Supplementary file 1 — Additional file 1: Table S1. Bacterial strains and plasmids used in this work. [file 40659_2022_377_MOESM1_ESM.docx]

| **Table S1. Bacterial strains and plasmids used in this work.** | | | |
| --- | --- | --- | --- |
| **Bacterial** | | | |
| **Strains** | **Relevant characteristic** | | **Source** |
| STm 14028s | Wild type strain of *Salmonella enterica* serovar Typhimurium (*S.*Typhimurium) 14028s | | G. Mora ATCC |
| Δ*micA* | *S.* Typhimurium 14028s Δ*micA::caf* | | McClelland M. et al (2014) |
| Δ*cyaR* | *S.* Typhimurium 14028s Δ*cyaR::caf* | | McClelland M. et al (2014) |
| Δ*oxyS* | *S.* Typhimurium 14028s Δ*oxyS::caf* | | McClelland M. et al (2014) |
| Δ*ryhB* | *S.* Typhimurium 14028s Δ*ryhB::aph* | | McClelland M. et al (2014) |
| Δ*hfq* | *S.* Typhimurium 14028s Δ*hfq::caf* | | C. Saavedra |
| Δ*ompX* | *S.* Typhimurium 14028s Δ*ompX::aph* | | C. Saavedra |
| Δ*ompX/*pBAD::*ompX* | *S.* Typhimurium 14028s Δ*ompX::aph* complemented with the plasmid pBAD, which carries the gene *ompX* | | C. Saavedra |
| STm 14028s/pGLO_*ompX* | *S.* Typhimurium 14028s/pGLO_*ompX* | | This work |
| STm 14028s/pGLO_*ompX* | *S.* Typhimurium 14028s/pGLO | | This work |
| *ompX::3xflag* | *S.* Typhimurium 14028s that carried the *3xflag* epitope on the *ompX* gene | | This work |
| TOP10 | *Escherichia coli* F- *mcrA* Δ(*mrr-hsdRMS- mcrBC*) φ80*lacZ*Δ*M15* Δ*lacX74 nupGrecA1*  *araD*139 Δ(*ara-leu*)7697 *galE*15 *galK*16 *rpsL*(StrR) *endA1* λ- | | Invitrogen |
| TOP10/ pGLO_*ompX* | *Escherichia coli* TOP10 that contains the pGLO_*ompX* plasmid | | This work |
| TOP10/pGLO | *Escherichia coli* TOP10 that contains the pGLO plasmid | | This work |
| **Plasmids** | | | |
| **Plasmid** | **Relevant characteristic** | **Source** | |
| pSUB11 | *bla*ori R6K *aph* FRT *3x-flag*, Kan^R^Amp^R^ | Uzzau et al. (2001) | |
| pGLO | P_BAD_, *bla*, ORF *araC,* ori pBR322, Amp^R^, ORF *gfp* | Bio-rad | |
| pGLO_*ompX* | pGLO_*ompX* plasmid that contains the *ompX* promoter of *S.* Typhimurium 14028s, this promoter comands the transcription of the *gfp* gene that encodes to the green fluorescence protein (GFP) | This work | |
