# Supplementary material for: Genetic regulation of the ompX porin of Salmonella Typhimurium in response to hydrogen peroxide stress
Source: Biol Res. 2022 Feb 22;55:8. doi: 10.1186/s40659-022-00377-3 (PMC8862304; doi:10.1186/s40659-022-00377-3)
Supplement: Supplementary file 2 — Additional file 2: Table S2. Primers used in this work. [file 40659_2022_377_MOESM2_ESM.docx]

| **Table S2. Primers used in this work.** | |
| --- | --- |
| **Primers** | |
| **Name** | **Sequence (5’🡪3’)** |
| RT_*talB*_R | ACTATGCGCCAGCTGAAGAT |
| RT_*talB*_F | TCGCTTTCCGCCAGTTCTTT |
| Prom_*ompX_-*1R | CCGACCGGCATCCTTTCTCCTGTCAAAAA |
| Prom_*ompX_-*375F | GGCAGCCTGAAAGACATGCAATTTTTTTCATAACCACCTCAA |
| ompX_FLAG_F*^a^* | TGACGTTGGCACCTGGATTGCTGGCGTAGGTTACCGCTTCgactacaaagaccatgacgg |
| ompX_FLAG_R*^a^* | TTTTTCACTAAAATAATGCACGTTTCAGGCAAAAAAAATCcatatgaatatcctccttag |
| *ompX*_RT_F | GGGCGTAGCGAATAAAATGA |
| *ompX*_RT_R | GAAGCCATAATCGCTGGTGT |

*^a^*: Primers that hybridize with the plasmid pSUB11 (lowercase) and having an extension of 40 bp of homology to a region of the target gene mutagenized by allelic exchange.
