# Supplementary figures and images for: Genetic regulation of the ompX porin of Salmonella Typhimurium in response to hydrogen peroxide stress
Source: Biol Res. 2022 Feb 22;55:8. doi: 10.1186/s40659-022-00377-3 (PMC8862304; doi:10.1186/s40659-022-00377-3)

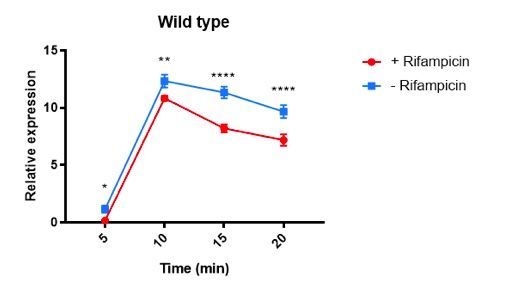

Supplement: Supplementary file 3 — Additional file 3: Figure S1. Effect of rifampicin on ompX mRNA under H2O2-induced stress in the wild-type strain. ompX mRNA levels were measured by qRT-PCR. When cultures reached an OD600 ≈ 0.4, a pulse of rifampicin (20 µg/ml) was added or not (+ RIF red line or –RIF blue line) to the culture at time 0 min. At each time point, RNA was isolated and subjected to qRT-PCR as described in “Materials and Methods”. Post-test Bonferroni: *p < 0.05, **p < 0.01 and *** p < 0.0001, (mean ± SD or SE?). [file 40659_2022_377_MOESM3_ESM.jpg]

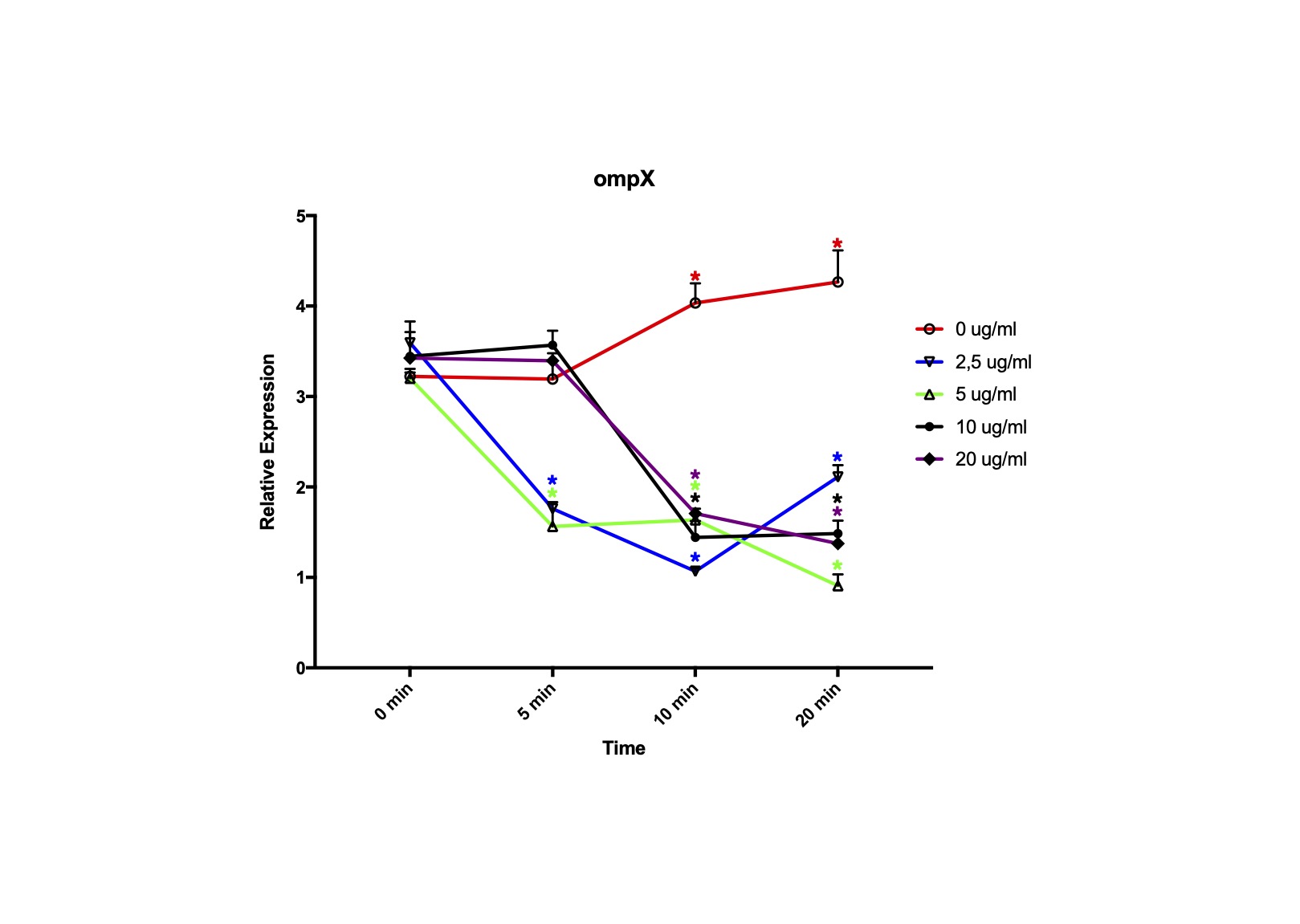

Supplement: Supplementary file 4 — Additional file 4: Figure S2. Effect of rifampicin concentration on ompX. The ompX mRNA levels were measured by qRT-PCR. When cultures reached an OD600 ≈ 0.4, a pulse of rifampicin (0, 2.5, 5 and 10 µg/mL), was added. At each time point, RNA was isolated and subjected to qRT-PCR as described in Materials and Methods. [file 40659_2022_377_MOESM4_ESM.jpg]

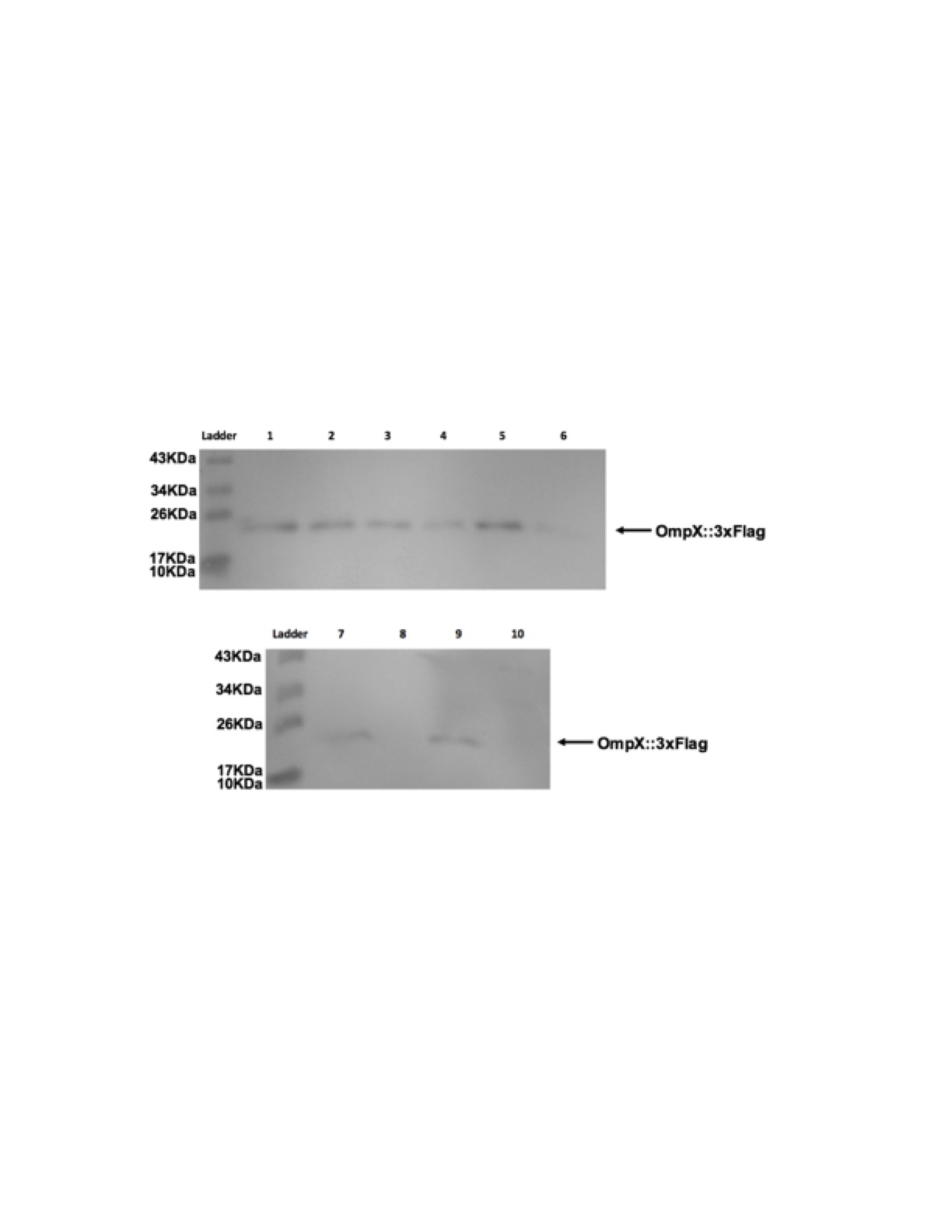

Supplement: Supplementary file 5 — Additional file 5: Figure S3. Production of OmpX::Flag protein in S. Typhimurium after exposure to H2O2. Outer-membrane fractions of each strain were isolated after culture under control or 2 mM H2O2 treatment (20 min in Lennox broth at 37 ºC). Lanes 1 and 2: ompX::3xflag control and H2O2-treated. Lanes 3 and 4: ΔcyaR ompX::3xflag control and H2O2-treated. Lanes 5 and 6: ΔmicA ompX::3xflag control and H2O2-treated. Lanes 7 and 8: ΔoxyS ompX::3xflag control and H2O2-treated. Lanes 9 and 10: Δhfq ompX::3xflag control and H2O2-treated. Total protein extracts (100 µg) were resolved by SDS-PAGE. The assay shown is representative of three biological replicates. [file 40659_2022_377_MOESM5_ESM.jpg]
